# Physiotherapy: a historical analysis of the transformation from an occupation to a profession in Brazil

**DOI:** 10.1590/bjpt-rbf.2014.0103

**Published:** 2015

**Authors:** Ana L. O. Oliveira, Everardo D. Nunes

**Affiliations:** 1 Escola Multicampi de Ciências Médicas do Rio Grande do Norte, Universidade Federal do Rio Grande do Norte (UFRN), Caicó, RN, Brazil; 2 Departamento de Saúde Coletiva, Faculdade de Ciências Médicas (FCM), Universidade Estadual de Campinas (Unicamp), Campinas, SP, Brazil

**Keywords:** physical therapy, history, professionalization, credentials, expertise, São Paulo

## Abstract

**Background:**

Analyzing the historical and social path of an occupation using the sociology of professions and the perspective of scientific knowledge promotes an understanding of the origin of physical therapy in Brazil and of discussions of the profession in its contemporary context.

**Objective:**

The aim of this paper was to discuss the professionalization process of physical therapy in São Paulo. The authors tried to analyze bath therapy, massage therapy, and physical therapy as occupations involving distinct *expertise* and as part of the group of occupations that evolved into the profession of physiotherapy in the first half of the twentieth century.

**Method:**

The analysis undertaken was a qualitative study based on an analysis of historical documents. Eighty-six professional records from the Service of Inspection of Professional Practice in the state of São Paulo and healthcare legislation from the 1930s and 1940s were analyzed.

**Results:**

The distinction between physical therapy practitioner and profession of physiotherapy can be seen by examining registration requirements for rank-and-file nurses with expertise in interactions; this distinction suggests the emergence of specialized expertise that was clearly a part of neither medicine nor nursing and contributed to expertise in physical therapy since the 1950s.

**Conclusion:**

The regulation of physiotherapy practices, the recognition of expertise, the accreditation of practical nurses by the State, and the institutionalization of a course for physical therapy practitioners in 1951 are key elements of the professionalization process for the physical therapy profession in Brazil.

## Introduction

Understanding occupations and professions as processes imbued with historical value can help one understand the adoption of certain therapeutic practices, including those used in specific historical contexts^1^, that have influenced the establishment of a profession. In this context, unregulated physical therapy practices in the 1930s and 1940s in São Paulo, Brazil led to the physiotherapy regulated profession, which is one of the 14 health-related professions regulated in Brazil.

The predominance of studies that form a part of physiotherapy knowledge based on the biomedical model is a cause for concern^2^ ^3^ since it theoretical orientation can hide some relevant social and historical aspects to understanding the profession today. This study aims to provide a critical and reflective socio-historical analysis of the knowledge and practices that shaped physical therapy as a distinguished healthcare profession in Brazil^4^ ^6^.

Expert knowledge, technical and intellectual recognition, accreditation, and professionalization of therapeutic practices are considered important for the social organization of work in the healthcare sector^7^ ^9^. The authors believe that the professionalization process of physiotherapy can be observed through the distinction between the characteristics of physiotherapy and physical therapy practices mentioned in the unprecedented descriptions of the work conducted by massage therapists, bath therapists, and unlicensed physiotherapists, who were licensed by the State in the first half of the twentieth century.

This paper examines the ways in which physiotherapy became established as a profession in Brazil. Unlike Anglo-Saxon language, physiotherapy and physical therapy in Portuguese language are not often used interchangeably. For the purpose of this manuscript, physiotherapy (*fisioterapia *) is understanding as a field of practice and formal knowledge. On the other hand, we defined physical therapy (*terapia física *) as a physical resource used by different occupational categories. In the paper we trace the shift from physical therapy as an occupational practice - once colonized by nurse practitioners and physicians - to physiotherapy through a range of mechanisms, including the development of labor organizations, economic rationalization, changes to the structure and socialization of professional expertise, and the development of accrediting institutions involved in the social configurations of Brazilian physiotherapy's professionalization history.

The organization of healthcare services in Brazil during this period was associated with measures taken during the first presidential mandate of Getúlio Vargas (1930-1945) who guided by the precepts of the labor movement, allegedly regulated occupational activities as part of his political strategy for modernizing the State using scientific advances in relevant areas^10^. On the basis of labor regulations from the 1930s and 1940s, the authors will use the theoretical framework of the sociology of professions^9^ ^11^ ^13^ and of scientific knowledge^14^ to define the concepts of occupation, profession, *expertise , interactional expertise , contributory expertise *, and accreditation with the aim of understanding the recognition of physiotherapy practices by the Brazilian State and by the medical community as part of the transformation of an occupation into a profession.

It is through the acquisition of *expertise * or the result of a socialization process that enables the development of a range of skills and specialized knowledge, that an occupational group pursues professionalization to create a set of practices, control labor activities, formalize teaching, receive recognition from peers and the public, become regulated and accredited by the State^11^, and promote social closure^9^ ^13^ in the labor market. These elements distinguish occupations from professions based on their distinct types of labor organization.

Occupations are characterized by the dissemination of knowledge acquired informally outside the official education system^11^ ^12^; the predominant learning strategy is enculturation, through which an occupation develops and absorbs knowledge through social relations called *interactional expertise* ^14^ Professions use the dissemination strategies of formal educational institutions and are characterized by the acquisition of the ability to perform a specialized practice through their own bodies of knowledge called *contributory expertise* 11 14 Both types of *expertise *, when officially recognized, guide the principles of the labor market into the organization and accreditation schemes provided by universities, associations, and/or the State^11^ ^13^

Physiotherapy was established as a profession in the state of São Paulo through the incorporation of occupational practices relating to physical rehabilitation, a process that is similar to the professionalization of occupational therapy (OT). However, the authors could not find records that allowed them to affirm the earlier existence of OT as an occupation. The authors did observe that the distinction between the two professions in the period analyzed was based on therapeutic interventions with a focus on technical training so that an occupational therapist was responsible for the rehabilitation of the upper body^15^, whereas physical therapy practice was responsible for the rehabilitation of the entire body. Despite the relationship between the two professions, in this study, the emergence of therapeutic practices considered characteristic of the professionalization of physiotherapy were addressed.

Therefore, the aim of this study was to identify physical therapy practices as evidence of the existence of an occupation that led to the professionalization of physiotherapy in the state of São Paulo. The authors believe that this study will help provide a historical context for physical therapy as a profession and promote a discussion about its scope, professional identity, and social role in the Brazilian healthcare system as a starting point for future investigations.

## Method

The method used for data collection was documentary research^16^ in the collection of records at the Professional Practice Supervision Service of São Paulo (Livros de Registros de Fiscalização do Exercício Profissional de São Paulo, LRFEP)^17^ from the National Health Department (Departamento Nacional de Saúde, DNS) and of federal and state laws in the state of São Paulo that were in force in the 1930s and 1940s^18-23^ The primary data analyzed were from the study called "History of Health Workers [História dos Trabalhadores da Saúde]" conducted in 2007 by the Public Health Memory Center of the Health Institute of the State Secretariat of Health of São Paulo.

The collection was preserved in the Documentation Center of the Butantan Institute at the Emilio Ribas Public Health Museum and included 119 professional licensing records covering the period between 1892 and 1978. According to Mott et al.^24^, this is an unprecedented collection, little of which has been explored by scholars dedicated to the analysis of professional training and the labor market in the healthcare sector. In this study, 86 records of practical nurses licensed as massage therapists, bath therapists, and physical therapists, which were occupations that made use of physiotherapy practices including physical and natural resources for medical treatment, were analyzed.

In 1951, with the institutionalization of the "Raphael de Barros" physiotherapy degree program for technicians and operators at the Faculdade de Medicina da Universidade de São Paulo (FM/USP ), São Paulo, Brazil, physiotherapy started to have its own registration records. These records extend to 1978, when the Federal and Regional Councils of Physical Therapy and Occupational Therapy (COFFITO/CREFITO) were permanently established.

The records were organized in Microsoft Excel 2007 spreadsheets and contained data on nine factors: name; gender; occupational category; date, city, state and country of birth; legislation in force at the time of registration; and year of registration (Table 1).

The profile description of a practical nurse and the differentiation between the terms "physical therapy practices" and "physiotherapy" will be addressed to provide a historical context for the institutional disputes over therapeutic resources, which, since 1969, have become part of the physical therapy profession.

## Results and discussion

Physical therapy practice and Physiotherapy are a healthcare practice that use physical and natural resources such as fresh or salt water, heat, cold, electricity, massage, and exercise as therapeutic resources. In Brazil the former is regarded as an occupation and the latter as a professional category, and they are differentiated by labor organization, rationalization, the structure and socialization of *expertise *, and the types of accreditation institutions involved in their social configurations.

Therefore, the authors consider physical therapy practice to include the activities of massage therapists, bath therapists, and physiotherapists practitioners in the 1930s and 1940s, and physiotherapy the set of practices accepted by the biomedical community between 1951 and 1969, when the physiotherapy degree program for technicians and operators was created at FM/USP, and the profession began to be regulated, respectively (Table 2).

This distinction is justified by the notion of a profession as a historical construct associated with specific social contexts that involves using a specific *expertise * to perform a set of social tasks that are used by members of the same professional or occupational category^9^ ^11^ Although physiotherapy was legally recognized as a profession in 1969^25^, the forces that drove the accreditation of physiotherapy *expertise * should be discussed.

### Physical therapy practices as *expertise*

In the 1930s and 1940s in Brazil, the Getúlio Vargas government played an important role in the organization of the social division of labor by creating a general system of laws aimed at regulating occupations and professions in the healthcare sector^11^ ^26^. This measure may be considered in the context of the accreditation of physical therapy nurses as a hallmark of the creation of physiotherapy. In this context, the role of the State in this period in generating reflections on the area of interest in this study cannot be ignored.

**Table 1:** Nursing occupational categories of bath therapists, physical therapy nurse, masseuses, and bath masseuses by gender, age and country of birth, 1930s-1940s.

Variable	Nursing occupational category		
	Bath therapists	Physical therapy Nurse	Masseuses	Bath-masseuses	Total n=86
	n= 6	n=2	n=74	n=4
Gender
Female	3	1	26		30
Male	3	1	48	4	56
Age
22-30 years			7		7
31-40 years	2	1	25		28
41-50 years	2	1	25	2	30
51-63 years	2		15	2	19
Not reported			2		2
Country of birth
Brazil	1	1	28	2	32
Other	5	1	44	2	52
Not reported			2		2

Source: Registry Books Collection of the Professional Practice Surveillance of São Paulo (LRSFEP), Emilio Ribas Museum, SP, Brazil^17^.

**Table 2:** Distinctions between physical therapeutic practices and physical therapy at two times, including the type of work, credentials, credentialing body, type of socialization or training, type of expertise, and therapeutic techniques.

	Physical therapy practice	Physiotherapy
Time Period	The 1930s and 1940s	The 1950s and 1960s
Work Type	Occupation	Occupation or/and Profession
Credential	bath therapists, physiotherapy nurses, masseuses	*Physical Therapy Technician* or *Assistants *
Credentialing Body	State	State Higher Education Institution
Socialization or Training	Informal (Practice - "on the job" training)	Formal (Institutionalized)
Expertise Type	Interactional Expertise	Contributory Expertise
Therapeutic techniques	Massage Bath Therapy Therapeutic Exercise Medical Electricity Heat/Cold Therapy Light Therapy	Massage therapy Hydrotherapy Kinesiotherapy Electrotherapy Thermotherapy Phototherapy

The strategies used by the State^11^ are similar to those present in the bureaucratic model discussed by Weber^27^. In this model, the State is regarded as an agent of political domination that has a rational-legal nature. In the healthcare sector, the regulation of certain occupational and professional categories, including unlicensed practical nurses, reflects this model, which is based on the rationalization of therapeutic practices and on the use of mechanisms of recruitment and qualification to leverage the professionalization of distinct occupational groups^28^ ^29^.

Rationalization is considered the subjection of social spheres to the criteria of rational decision-making, a characteristic of the institutionalization process and of the conversion of technique into science^27^. Technique and science come to exist as institutions and as part of the public discourse when they penetrate institutional spheres through their *expertise *.

The LRFEP allowed the recognition of therapeutic techniques specific to members of a given occupational category, unlicensed practical nurses. By describing 86 massage therapists, bath therapists, and physiotherapists practitoners as having *interactional expertise * regulated by the State, the authors propose an analytic framework within a broader historical context to explain the transformation of physiotherapy practices into *expertise * specific to physical therapy as a profession. It is notable that, although physiotherapy nurses were not considered in the legislation analyzed, two nurses, a Brazilian and a German, were registered under this title, suggesting the existence of a category recognized for its members' use of a combination of resources in their practices.

Therefore, *expertise * historically manifests itself within a network of social and political relationships that are elements of the labor structure founded on the rationalization of therapeutic practices based on qualifications, merit, and competence that allow expertise to be recognized as a prerequisite for professionalization. However, *expertise * is a result not only of special cognitive processes but also of social practices, of the context in which it operates, and of disputes over power. It has two types: *interactional expertise * and *contributory expertise* 14.

*Interactional expertise* is the ability to perform tasks based on an accumulation of knowledge that was informally acquired and incorporated, by means of experience or wisdom, through enculturation in a scientifically established field. In addition, this type of expertise should enable its practitioners to add knowledge to their areas of practice, - i.e., allowing *contributory expertise.* Therefore, physiotherapy practices are considered, in this context, as the outline of a field within other fields- e.g. medicine and nursing-and inserted into a specific historical context.

Important events, including the two World Wars, the polio epidemic, occupational accidents, and the process of urbanization and industrialization in São Paulo, created a social demand for the unique technical resources of physical therapy practices. In this context, the technological advances in the field of rehabilitation and the discovery of the therapeutic potential of physical and natural resources^4^ ^30^ ^32^ led to the emergence of an *expertise * that was disputed by medicine and nursing. Despite the high degree of *interactional expertise *, the knowledge of these *experts *-non degree nurses-was not necessarily formally recognized.

One example is that of Moreira^33^, who described the treatment of people affected by polio and described massage, electric currents, active and passive exercises, and exercises performed in pools and hot baths as important therapeutic resources for rehabilitation. By arguing that such treatment should be performed by nurses with experience in these therapeutic practices, Moreira^33^ showed that the physical therapy nurses who, in the absence of their own professional field, were influenced by the knowledge that originated from exchanges with other countries.

The migrations in Brazil during the 1930s and 1940s influenced the creation of an occupational category for physiotherapy nurses. This fact is evident when comparing the number of foreigners (52) and Brazilians (32) registered with the LRFEP and can be explained by viewing the exchange of *expertise * between European countries and Brazil as one of the political strategies employed by Vargas in this period to stimulate the modernization of the country and, consequently, of national science^34^.

The city of São Paulo was home to many foreign workers, and the average age of the registered workers, 42 years, suggests that practitioners used physical therapy practices before the accreditation requirement, demonstrating that these practices existed before the teaching of physiotherapy was institutionalized.

Physical therapy *expertise * could be define as an occupation defining by Freidson^11^ as a specializations based on experience and practical training guided by professions with formal education. Therefore, the incorporation of these occupational techniques and therapeutic methods into the social division of labor in the healthcare sector was disputed by the institutionalized professions.

This organization of labor, which was regarded as the result of interactions and social construct processes, promoted the dissemination of physical therapy practices in the daily practice of professionals during the 1930s and 1940s as an important technique for rehabilitation and healthcare in the context of several professional categories. By gaining strength and technical and intellectual recognition, physiotherapists became accredited by the State as licensed practical nurses.

### The State as an accrediting institution

As previously mentioned, the Vargas government acted as the accrediting institution for the regulation of health practices^26^ ^28^. The regulation of expert knowledge, which has been typical of modern Western societies, served as a strategy for reorganizing labor in the healthcare sector based on the selection of a certain type of *expertise * as a prerequisite for the implementation of social policies in a stratified system of occupations. This decision led the State to professionalize certain practices, including physical therapy practices.

By observing the political processes by which those practices were incorporated into medicine and nursing, the authors can confirm that the existence of physical therapy *expertise * stimulated the development of a plan for the professionalization of this group of occupations starting in 1951. Massage therapist, bath therapist and physiotherapist nurses, finding fertile ground for their work, even without autonomy, were able to monopolize occupational practices linked to the idea of expert knowledge through accreditation by the State. This exclusive therapeutic practice, which became a part of the field of ​​knowledge, led to social closure in the social division of labor after the regulation of the profession began in 1969^9^ ^11^ ^13^.

The occupation of physical therapy nurses, who had an *interactional expertise* that was politically recognized and socially necessary, was considered by the state legislature in São Paulo and officially became an occupational category with Decree No. 20.931 on January 11, 1932^18^. This Decree also established the regulation and supervision of the practices of physicians, dentists, veterinarians, pharmacists, midwives, and nurses. By requiring that optometrists, pharmacy practitioners, massage therapists, and bath therapists must be supervised "[...] and could only practice their profession if they proved to have the proper licensing at the discretion of the health authority [...],"^18^ the State added physiotherapy practices to the list of the country's accredited therapeutic resources.

Accreditation did not guarantee that these professional activities could be practiced indiscriminately because "[...] physical therapy, orthopedics, and beauty institutes and commercial establishments with medicinal showers or baths as well as those that use electrotherapy, radiotherapy, and post-surgery exercises should operate under the responsibility and technical supervision of doctors or pharmacists."^18^ Therefore, physiotherapy practices were not technically autonomous at this time.

However, the legalization of these practices, which was obtained through *interactional expertise *, showed recognition of the value of knowledge and of scientific knowledge in the healthcare sector in Brazil^35^. The publication of Decree No. 23.774 on January 22, 1934^19^ highlighted this fact because nurses who submitted certificates proving that they had more than five years of experience were given the title of "practical nurses" by the DNS after completing a qualifying examination. The demonstration of *expertise * became an obligatory condition of the work of unlicensed nurses in the healthcare sector.

This requirement of the state of São Paulo underlined the power relationship between medicine and nursing when, in 1938, State Decree No. 9.707^20^ established a requirement that doctors be present as directors and professors of nursing services that trained and accredited qualified professionals in regular and specialized programs. At that time, medicine and nursing were distinguished by the role of physiotherapy *expertise. * The former had the dominant *expertise * needed to teach and oversee the practice of nurses, and the latter was responsible for delivering therapeutic practices.

The following year, unlicensed nurses were recognized by state law with the approval of Decree No. 10.068 in 1939^21^, which considered massage and bath therapy as therapeutic resources of nurses. Therefore, nursing activities became those activities performed by licensed nurses as well as by midwives, massage therapists, bath therapists, and chiropodists/podiatrists. Furthermore, to be accredited, practical nurses were evaluated by committees composed of doctors and licensed nurses.

The State became the accrediting institution that was responsible for the regulation, supervision, and definition of the occupational activities of each group, thereby controlling these professions and occupations. Massage therapy was accepted as the primary physical therapy practice; the evidence for this fact is that massage therapists accounted for 86% of the 74 registrations in this period.

Gender differences in the division of labor in the Brazilian healthcare sector were driven by the distinction between care and cure; the former was predominantly conducted by women and the latter by men. This fact may explain the greater emphasis on scientific power to the detriment of the practice of care, which was considered a physical therapy practice and currently an integral part of physiotherapy's "therapeutic arsenal". An example of this distinction is found in the formalization of the teaching of physical therapy practices in nursing by Decree-Law No. 8.778 in January 22, 1946^22^. Physical therapy *expertise *, when considered as a resource for human care, became a discipline in the area of knowledge known as "the art of nursing," which used resources such as "[...] hot and cold packs, compresses, poultices and suction cups, hot and cold therapies [...]"^35^ in nursing education.

The contradiction between the institutionalization of this discipline in nursing, which was a profession primarily taken up by women, and the predominance of male physical therapy nurses registered in the LRFEP (n=56) demonstrates the clash between the "art" and "science" of in the healthcare sector. The former was directed toward care and the latter was directed toward the therapeutic efficacy advocated by Western scientific medicine.

The scenario changed in 1949 when the content of nursing and nursing assistant degree programs was regulated through Decree No. 27.426^23^, and physical therapy practices developed more rational boundaries. Part of the content to be disseminated by official schools was physical therapy *expertise *, which was institutionalized in the teaching of "[...] clinical orthopedic clinic, physical therapy, and massage therapy [...]"^35^ offered in the second year of the nursing program.

At that time, the competition for physical and natural resources between the institutions of medicine and nursing intensified. The predominance of medicine in this historical period is clear because physiotherapy degree program for technicians and operators were established in 1951, and physical therapy practices began to be taught by physicians at FM/USP instead of being part of the nursing degree program. The training of technicians lasted until 1967 when the physiotherapy and occupational therapy degree programs, still held at FM/USP, began to be regulated through Ordinance GR 347^36^.

From that moment, physiotherapy began to define its own scope of practice while remaining under the tutelage of medicine. Although it was incipient, the professionalization process and, consequently, the possibility of the *interactional expertise * of physiotherapy practices transforming into *contributory expertise, * was initiated.

Social changes and the elements associated with them, including the establishment of an occupational group such as physiotherapy, were slow processes that required reflective critical attention. Physiotherapy is a professional category with a legacy related to its practical and theoretical knowledge. Reflections of its historical context should be reconsidered to allow an understanding of the professional identity of the contemporary physical therapist to be an essential condition for facing the current challenges of the profession.

## Final considerations

The development of an occupation or profession is a historic variant of the process of organizing a society and creating a monopoly on opportunities. In this study, the authors have sought to demonstrate that opportunities, social needs, disputes over monopolies of certain practices, interactions between professional fields, and State accreditation were all important factors that helped physical therapy practices become a type of *expertise * accepted as an occupation in São Paulo in the 1930s and 1940s.

The activities performed by physical therapy nurses made it possible for this occupational category to acquire legal status in 1939 in São Paulo. The *interactional expertise * of physical therapy practices was evidence for the existence of tacit knowledge, which was foundin the records of licensed practical nurses available from the LRFEP.

Using accreditation as a measure of social control and social closure was the political strategy chosen to regulate the healthcare practices adopted by unlicensed occupational groups. Therefore, the State was the sovereign accrediting institution in the 1930s and 1940s and based the process on the rational-legal organization model by valuing expert and scientific knowledge as a basis for organizing labor and defining the activities of occupational groups.

In Brazil this occupational closure promoted the pre-professionalization of physiotherapy, which began a new phase in 1951, when physical therapy practices were disseminated through the Raphael de Barros Physical Therapy degree program for Technicians and Operators at FM/USP. Establishing themselves as holders of *interactional expertise *, physical therapy nurses encouraged the professionalization of physiotherapy through the recognition and accreditation of their knowledge. Understanding this recognition by medicine and nursing, in addition to the State's accreditation of practical nurses, helped clarify the means by which this occupation gained momentum in its fight for professionalization. Therefore, in the 1930s and 1940s, physiotherapists had not only the social authority to perform their activities but also permission to establish a new occupational category in the face of the existing professional groups, i.e. medicine and nursing, in the healthcare sector in São Paulo.

By recognizing *interactional expertise * as evidence for the transformation of an occupational group into a profession, one may assume that bath therapists, massage therapists, and physical therapy nurse were part of the group involved in the professionalization of physiotherapy. After the 1950s, *interactional expertise * was differentiated and organized so that it became *contributory expertise *, which enabled physiotherapy to become a proper profession in the late 1960s.
